# A plausible universal behaviour of earthquakes in the natural time-domain

**Published:** 2004-06-01

**Authors:** Haruo Tanaka, Panayiotis A. Varotsos, Nicholas V. Sarlis, Efthimios S. Skordas

**Affiliations:** *) Earthquake Prediction Center, Tokai University, 3-20-1, Shimizu-Orido, Shizuoka 424-8610, Japan; **) Solid State Section, Physics Department, University of Athens, Panepistimiopolis, Zografos 157 84, Athens, Greece; ***) Solid Earth Physics Institute, Physics Department, University of Athens, Panepistimiopolis, Zografos, 157 84, Athens, Greece

**Keywords:** Natural time, critical dynamics, seismicity, universality

## Abstract

In previous papers, we showed that in the new time-domain, called natural time: 1) the power spectrum of an SES-activity exhibits properties of *critical* phenomena and the power spectra of the local seismicity subsequent to an SES-activity become the same as that of the latter just before the main shock, 2) the power spectra of regional and long-term seismicity in both Greece and San Andreas fault system also exhibits features of *critical* phenomena. Here we show that the *same* can be found for the earthquakes in Japan. The “power spectra” in natural time of the seismicities in all the three areas fall on a *universal* curve.

## Introduction

The analysis of seismicity as well as that of Seismic Electric Signal (SES) activities (e.g., refs. 1), 2)) has always been carried out in the conventional time-domain. It has recently been shown,3)–7) however, that the use of a new time-domain, termed natural time *χ*, exhibits several distinct advantages. The use of this new time-domain was motivated by studies on complex systems and critical dynamics.3)

The *natural time χ*_k_ = k/N serves as an index3) for the occurrence of the kth event in a time series comprised of N events such as SES activity and associated seismicity. For the analysis of SES, we considered the time evolution of the pair of two quantities, i. e., (*χ*_k_, Q_k_) where Q_k_ denotes the duration of the kth signal. We introduced a continuous function F(*ω*) defined as follows:

[1] $$\text{F }(\omega )=\sum_{\text{k}=1}^{\text{N}}{\text{Q}}_{\text{k}}\;\text{exp}\left(\text{i}\omega \frac{\text{k}}{\text{N}}\right)$$

where *ω* = 2*πφ*, and *φ* stands for the *natural frequency*. Dividing by F(0), we normalise F(*ω*):

[2] $$\Phi (\omega )=\frac{\sum_{\text{k}=1}^{\text{N}}{\text{Q}}_{\text{k}}\;\text{exp}\left(\text{i}\omega \frac{\text{k}}{\text{N}}\right)}{\sum_{\text{k}=1}^{\text{N}}{\text{Q}}_{\text{k}}}=\sum_{\text{k}=1}^{\text{N}}{\text{p}}_{\text{k}}\;\text{exp}\left(\text{i}\omega \frac{\text{k}}{\text{N}}\right)$$

where

[3] $${\text{p}}_{\text{k}}={\text{Q}}_{\text{k}}/\sum_{\text{k}=1}^{\text{N}}{\text{Q}}_{\text{n}}.$$

We can regard, in analogy to the probability theory, p_k_ and *χ*_k_ = k/N a probability value and probability variable respectively.

Then, we defined Π(*ω*) by [4] below. This may be regarded as a kind of normalised power spectrum

[4] $$\Pi (\omega )={\left|\Phi (\omega )\right|}^{2}.$$

For natural frequencies *φ* less than 0.5, Π(*ω*) or Π(*φ*) reduces3) to the characteristic function for the probability distribution p_k_ in the context of probability theory. Therefore, in what follows, we will be discussing on the properties of Π(*ω*) or Π(*φ*) in this range of *φ*.

Motivated by the piezo-stimulated currents’ model1),2) for the SES generation, i.e., SES is generated by a *cooperative* reorientation of the electric dipoles within crystals of rock-forming minerals upon approaching a *critical pressure*, our theoretical analysis 3) based on the theory of *critical* phenomena (see also refs. 4) and 10) and references therein) showed that the following relations should hold for an SES-activity:

[5] $$\Pi (\omega )=\frac{18}{5\omega^{2}}-\frac{6\text{cos}\omega }{5\omega^{2}}-\frac{12\text{sin}\omega }{5\omega^{2}}$$

where *ω* (=2*πφ*) denotes the angular *frequency in natural time*. Expanding eq. [5] around *ω* = 0, we get

[6] $$\Pi (\omega )=1-0.07\omega^{2}+\cdots .$$

This implies3) that the variance of *χ* is:

[7] $$\kappa_{1}=<\chi^{2}>-<\chi {>}^{2}=0.07.$$

According to the probability theory, the moments of a distribution and hence the distribution itself can be determined once the behaviour of the characteristic function of the distribution is known around zero. Our earlier studies 3),4) showed that actually all SES activities exhibit a *universal* behaviour obeying the relations [5] – [7], whereas these relations are *not* obeyed5),6) by artificial noises or ionic current fluctuations in biological membrane channels, even when they are indistinguishable in conventional time. This indicates that among those investigated only the observed SES have the properties compatible with those of the critical phenomena.

## Natural time-domain analysis of seismicity

The analysis of the seismic activity in the natural time-domain can be made similarly. In this case, however, instead of Q_k_, we ascribed the corresponding seismic moment M_0_ to the kth seismic event3),8)–10) and the time evolution of the pair (*χ*_k_, M_0 k_) was investigated. Analysis of seismicity in the natural time-domain led to the following two major conclusions. It is the aim of the present study to investigate whether these two conclusions hold for Japanese earthquakes or not. If so, they may be regarded as universal.

1) Analysing the seismicity in a candidate area which can be estimated from the observed SES data, the following interesting property has been shown3): If we set the natural time for the seismicity zero at the initiation time of the concerned SES-activity, we can form time series of seismic events in natural time for various time windows as the number of consecutive earthquakes N increases. When we compute Π(*φ*) for each of the time windows, we found that, in the range 0 < *φ* ≤ 0.5, it approaches, as N increases from 6 to some value less than 40, to that given by eq. [5], namely that of the SES-activity. Interestingly, this coincidence of Π(*φ*) of seismicity and SES-activity happens only a few days before the anticipated main shock, thus providing an effective way to make time prediction of a major earthquake much more precise than before. This investigation so far has been possible3),8)–10) only for Greece because no SES-activity data has been available from other areas.

When earthquakes occur in the way described above, we call the process “single correlated process”. When the next main shock occurs within a short time, however, the situation becomes complex. As mentioned above, to observe the approach to the critical stage, i. e. the state of Π_seis_(*φ*) = Π_SES_(*φ*) = Π_theoretical_(*φ*), where the subscripts indicate seismicity, SES-activity and the theoretical, it was found necessary to sample seismic data before the main shock for an appropriate window length *l* which includes certain number of events (cf. it depends, of course, on the magnitude threshold) e.g. N = 40. After the main shock, there will be aftershocks for some time. If the time window to investigate the next main shock happens to overlap any part of the time windows and the aftershock period of the preceding earthquake, Π_seis_(*φ*) for the second earthquake would be seriously contaminated by un-related events.

2) The long period properties of the seismicity in wider regions also exhibit some characteristics associated with eqs. [5] – [7]. Such a study was performed in refs. 3) and 8) for seismic areas in Greece and in San Andreas fault system and the results are summarized below.

**Fig. 1:**
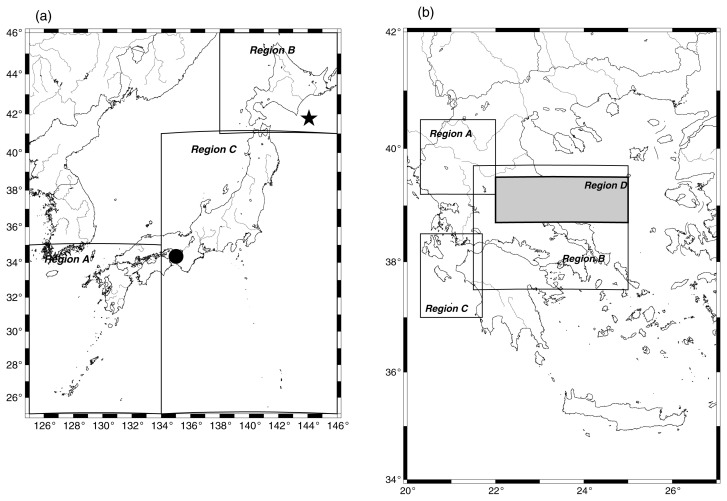
Maps of the regions in Japan (a) and Greece (b) in which the seismicity has been studied. The solid dot and the asterisk in (a) show the epicenters of Kobe and Off Tokachi earthquakes, respectively. For the San Andreas fault system, the coordinates for the region investigated is discussed in the text.

Varotsos *et al*.8) analyzed the seismic data of the whole Greek region, using the catalogue by the National Observatory of Athens, (NOA) for the period 1966–2001. First, calculation of the power spectra Π(*φ*) was made for an event taking time windows for 6 to 40 consecutive events, i. e., taking N = 6, 7, …, 39, 40. And second, this process was performed for all the events by scanning the whole catalogue. The lengths of the windows were chosen to cover the limits of the number of the earthquakes—after SES-activity—through which the coincidence of Π(*φ*) of seismicity and SES was reached as mentioned in 1). The choice of the precise value of the upper limit was not found decisive, because practically the same results were obtained8) even if the number of consecutive events was changed from 6–40 to 6–100. Then, the values of the observed probability P[Π(*φ*)] versus Π(*φ*) have been plotted. An inspection of the results showed that the local maxima of the curves, each one of which was drawn for a certain *φ*-value, correspond to the Π(*φ*) values that lie very close to those predicted from eq. [5]. Essentially the same was found3),8) for [1] each of the four smaller regions in Greece (Fig. 1(b)) surrounding the main shocks that occurred during the period 1988–2001 and [2] the San Andreas fault system. For the San Andreas area, we used the catalogue available from http://www.data.seec.org:3128/ftp/catalogs/SCSN/, for the period 1973–2003, within the area N_32_^37^ W_122_^114^.

## The present study

For Japan, we used the JMA catalogue for the period 1967–2003 within N_25_^46^ E_125_^146^ and employed the following approximate formulae obtained from a fit to Fig. 5.3 of ref. 11): M_w_ = 0.701M_J_ + 1.47 for M_J_ < 5, M_w_ = 0.916M_J_ + 0.40 for 5 ≤ M_J_ < 6, M_w_ = 1.07M_J_ – 0.509 for 6 ≤ M_J_ < 7.3, M_w_ = 1.345M_J_ – 2.56 – 0.0472/(M_J_ – 8.3) for 7.3 ≤ M_J_. Then the relation M_0_ ≈ 10^1.5 Mw^ has been used to obtain the values of moment M_0_.

*Short-term evolution of* Π*(φ) of local seismicity before major earthquakes: The Kobe, M(JMA) 7.2, 1995 and Off Tokachi, M(JMA) 8, 2003 earthquakes* (See Fig. 1a for their epicenters). Since no SES-activity data are available to us for these earthquakes, trial computation of Π(*φ*) was made for many time windows before them. Details will be published elsewhere, along with the results for the activity in 2000 in Izu Island region for which SES activity data have been published. 12) The main results for Kobe and Off Tokachi earthquakes could be summarized as follows:

1. Kobe earthquake. Investigating the seismicity (with magnitude threshold M(JMA)≥2.5) within the area N_32.5_^36.5^ E_132.5_^136.5^ and starting the Π(*φ*)-calculation from October 30–November 3, 1994, we find that the computed Π(*φ*)-value coincided to that of eqs. [5] to [7] on January 15–16, 1995, i.e., 1 to 2 days before the main shock (N = 70 or 64 for October 30 and November 3, respectively). If the calculation is repeated for a smaller area around Kobe, i.e., N_33.36_^35.36^ E_134.02_^136.02^, the result is changed only slightly, i.e., the aforementioned coincidence occurred on January 14–16, 1995 (N = 52 or 47 for October 30 and November 5, respectively).
2. Off Tokachi earthquake. Investigating the seismicity (with magnitude threshold M(JMA)≥3.0) within the area N_38.78_^44.78^ E_141.08_^147.08^ and starting the Π(*φ*)-calculation on July 10, 2003, the computed Π(*φ*)-value coincided to that of eqs. [5] to [7] on September 22–23 (N = 166–167) as well as on September 24–25, 2003 (N = 171–174). The calculations repeated for an appreciably smaller area N_40.78_^42.78^ E_143.08_^145.08^ (which is comparable to the small region investigated around Kobe) did not lead to *any* coincidence even if our calculation was started significantly earlier, i.e., since April 1, 2003. This could be understood in the context that the rupture length of the Off Tokachi earthquake (and hence the corresponding preparation area) was considerably larger than that of the Kobe.

### Long-term regional seismicity

In the following, we use, instead of P[Π(*φ*)] defined above, the corresponding probability density labelled p[Π(*φ*)], i.e., $\text{P}\left[\Pi (\varphi )\right]=\int_{\Pi (\varphi )-ɛ/2}^{\Pi (\varphi )+ɛ/2}\text{p }[\xi ]\;\text{d}\xi$, where *ɛ* denotes an infinitesimal small positive number. (Cf. in our earlier studies3),8)–10) for Greek and San Andreas earthquakes we used P[Π(*φ*)]).

**Fig. 2:**
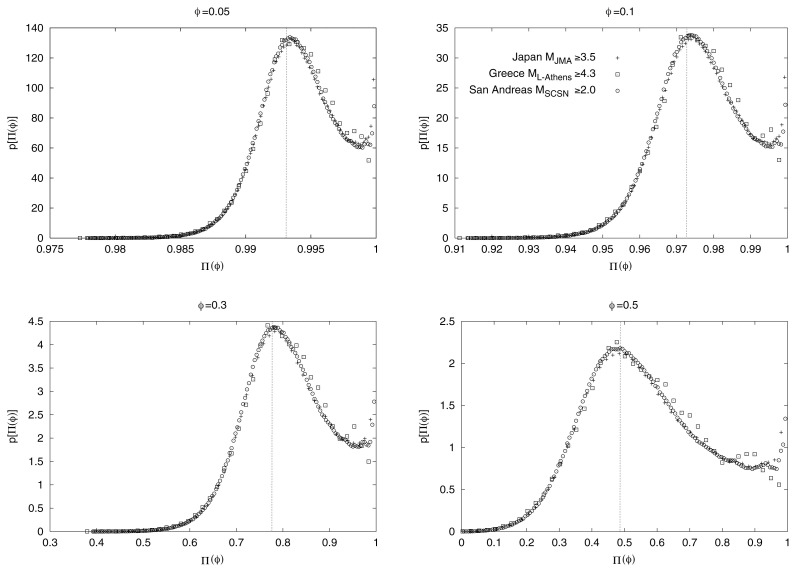
The observed probability density p[Π(*φ*)] versus Π(*φ*) for the seismicity in Japan, Greece and the San Andreas fault system for each of the following *φ*-values: *φ* = 0.05, 0.1, 0.3 and 0.5 for the period mentioned in the text. The dotted vertical bar shows, in each plot, the Π(*φ*)-value obtained from eq. [5].

The values of the observed p[Π(*φ*)] versus Π(*φ*) are given in Fig. 2 for the seismicity in the Japan area during the period 1973–2003 within the area N_25_^46^ E_125_^146^. For comparison, the results for the whole Greek area during the period 1966–2001 with ML ≥ 4.3, and for the San Andreas fault system during the period 1973–2003 within N_32_^37^ W_122_^114^ are also depicted. The values for all these three areas are given in the same figure for four *φ*-values: *φ* = 0.05, 0.1, 0.3 and 0.5. We find that the maximum value p_max_ of p[Π(*φ*)] in each plot lies at a value of Π(*φ*)-hereafter called Π_max_(*φ*)-very close to that predicted from eq. [5]; as shown by the dotted vertical lines drawn in each plot. This proximity may be understood, as already mentioned, in the following context: when sliding, the time window through the whole catalogue, the most probable value of Π(*φ*) is the one predicted from the theory of *critical* phenomena, i.e., eq. [5].

**Fig. 3:**
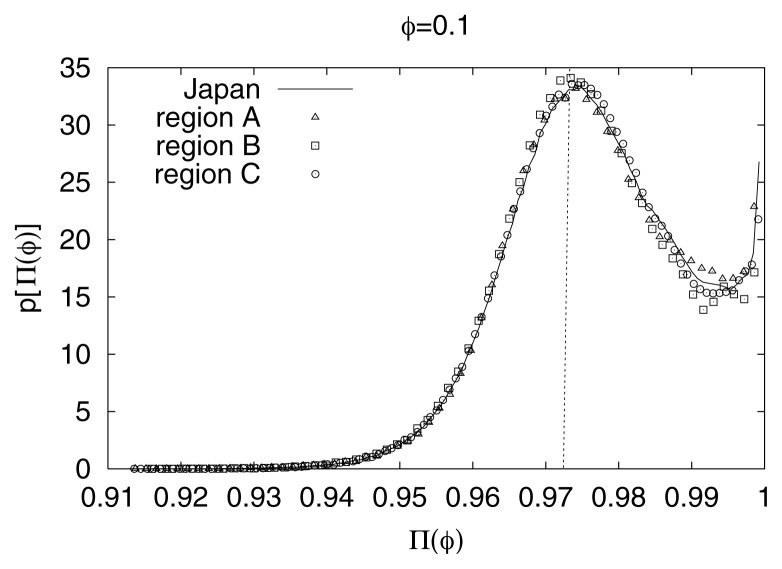
The observed probability density p[Π(*φ*)] versus Π(*φ*), for three different regions in Japan (see Fig. 1 a), i.e., A (triangles), B (squares) and C (circles), along with the results of the whole area of Japan for *φ* = 0.1.

**Fig. 4:**
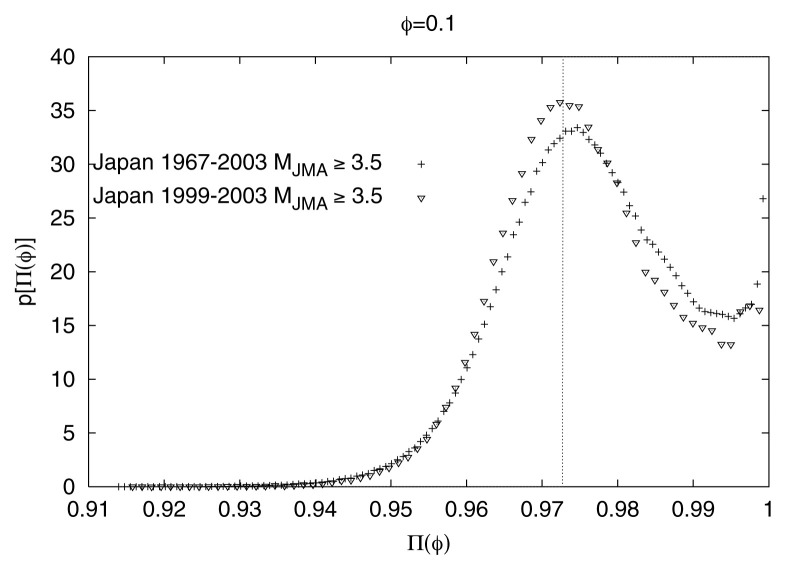
The value of Π_max_(*φ*) versus Π(*φ*) of the whole area of Japan for *φ* = 0.1 for the following two periods: (i) five years (1999–2003) and (ii) thirty seven years (1967–2003).

**Fig. 5:**
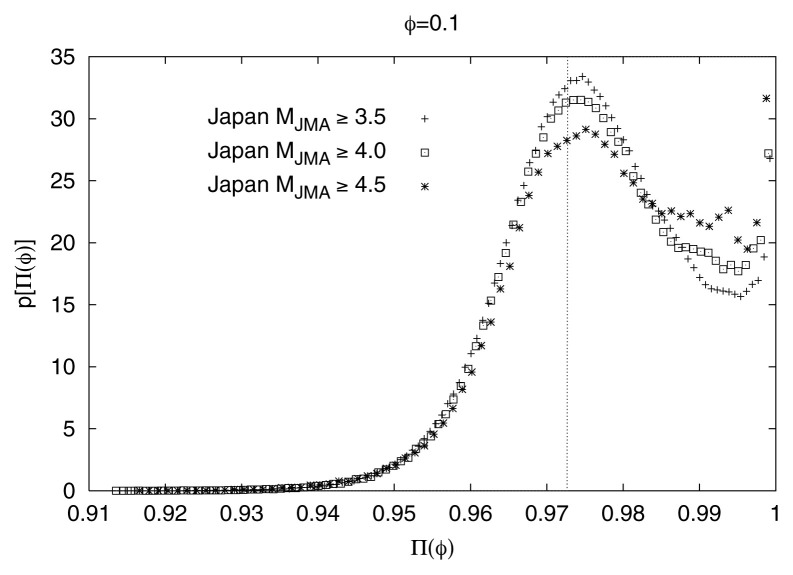
The observed probability density p[Π(*φ*)] versus Π(*φ*) in Japan, for *φ* = 0.1, for three magnitude thresholds. Note that the observed Π_max_(*φ*)-values lie very close to that (vertical dotted bar) predicted from eq. [5].

It has been confirmed that the main feature of Fig. 2 (i.e., the proximity of Π_max_(*φ*)-values with those predicted from eq. [5]) remains practically *invariant* upon changing either the seismic region or the time-period (i.e., *spatially* and *time invariant*) as well as the magnitude threshold. This can be seen in Figs. 3, 4 and 5, respectively for the Japanese areas. More precisely, in Fig. 3, we show an example of p[Π(*φ*)] for *φ* = 0.1 for the three different regions indicated in Fig. 1a, i.e., Regions A (triangles), B (squares), and C (circles), along with the results for the whole area of Japan. In Fig. 4, we show, as an example, that the value of Π_max_(*φ*) for *φ* = 0.1 for all Japan is not practically affected if we consider —instead of the 37 year period— a shorter time period of 5 years. Note that the observed Π_max_(*φ*)-value again lies very close to that (vertical dotted line) predicted from eq. [5]. Finally, Fig. 5 shows that the value of Π_max_(*φ*) for all Japan is not practically affected if we change the magnitude threshold.

**Fig. 6:**
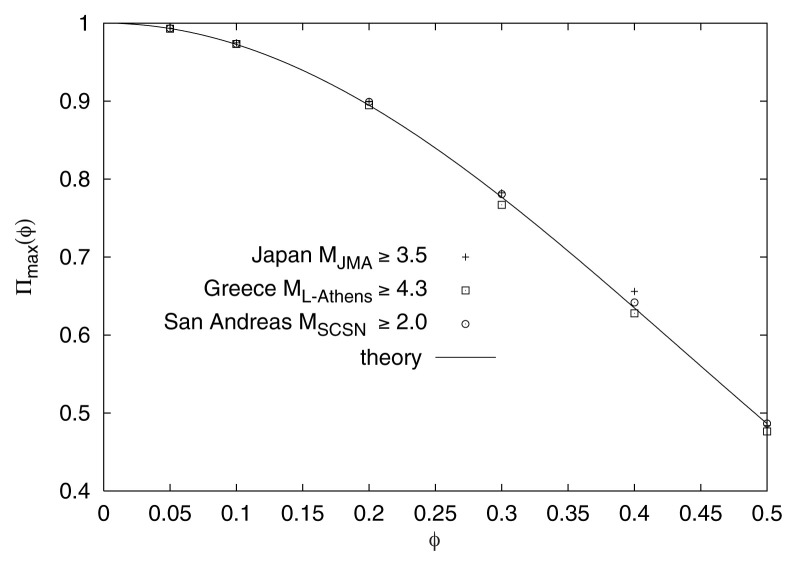
The Π_max_(*φ*)-values resulted from Fig. 3 versus *φ* for Japan (crosses), Greece (squares) and San Andreas (open circles). They scatter around the solid line drawn according to eq. [5].

The values obtained for Π_max_(*φ*) are plotted in Fig. 6 versus *φ*. Actually, the results for all the areas studied, i.e., Japan, Greece and San Andreas, the points are on the theoretical line drawn in the figure according to the theory of critical phenomena, i.e., eq. [5]. It is *striking* that the independent analysis of SES-activities in the natural time-domain led to the same theoretical *universal* curve.3),4)

**Fig. 7:**
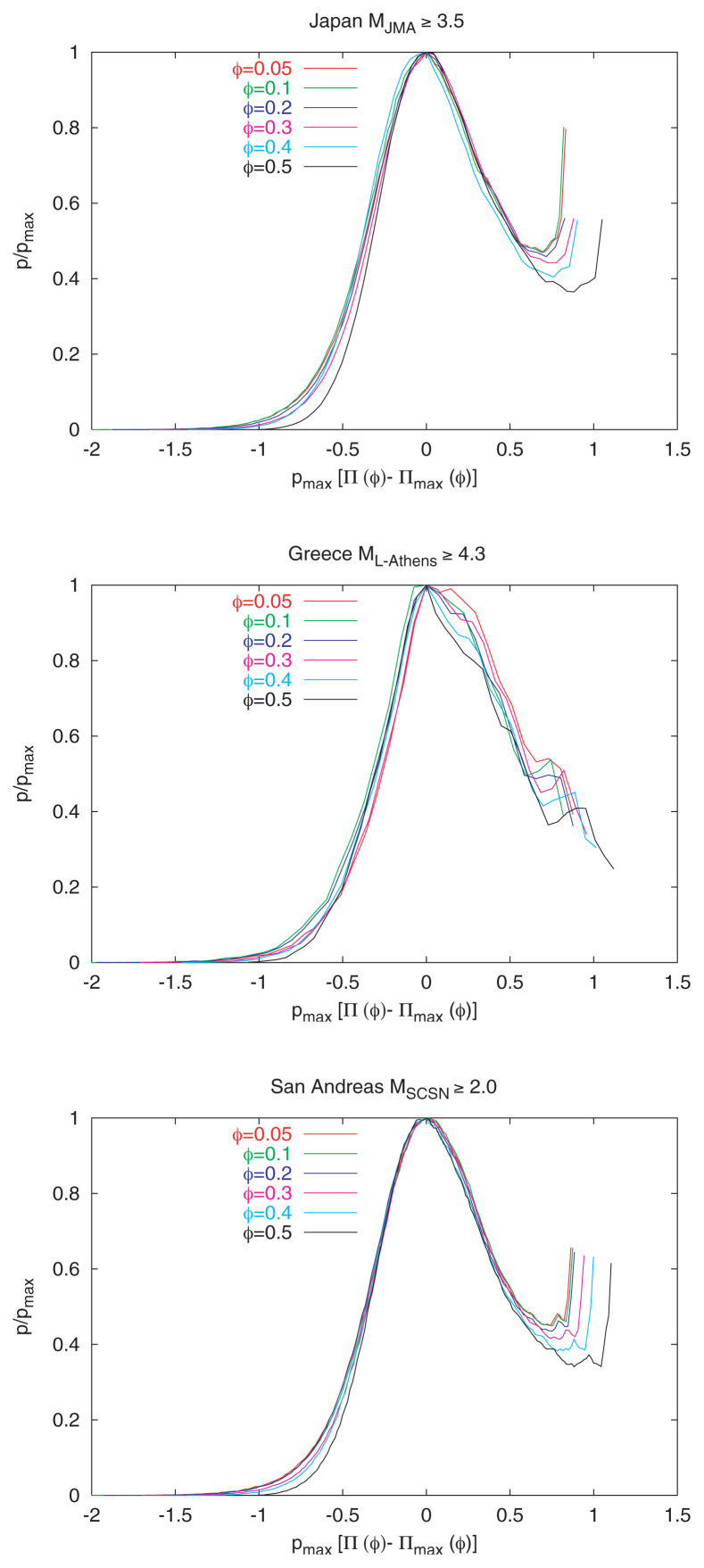
The p/p_max_ versus p_max_[Π(*φ*) – Π_max_(*φ*)] for each area separately, i.e., Japan (upper), Greece (middle), San Andreas (bottom).

**Fig. 8:**
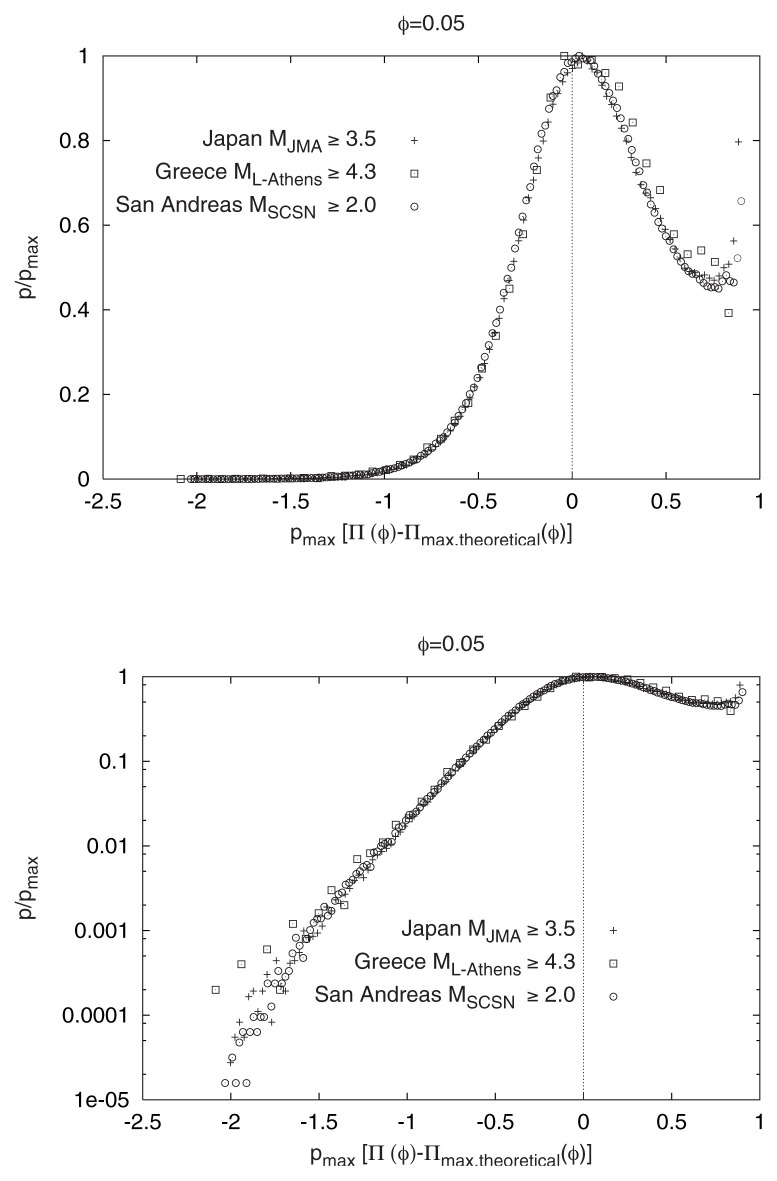
The reduced probability density p/p_max_ versus p_max_[Π(*φ*) – Π_max, theoretical_(*φ*)] for all the seismic areas investigated. Note that in this universal curve there are two segments which correspond to positive and negative values of the horizontal axis.

The four cases depicted in Fig. 2 correspond to the p[Π(*φ*)] versus *φ* relation for different *φ*-values. These can be reduced to almost the *same* curve, for each area separately, if we plot in Fig. 7 p/p_max_ versus p_max_[Π(*φ*) – Π_max_(*φ*)]. In Fig. 8, we plot, for *all* the seismic areas, the reduced probability density p/p_max_ versus p_max_[Π(*φ*) – Π_max, theoretical_(*φ*)], where the subscript “max, theoretical” denotes the value calculated from eq. [5]. All the data seem to fall on the same curve (*universal* curve). Both panels of Fig. 8 refer to the same quantities: in the upper panel, a linear-linear plot is given, while in the lower we turn it to a log-linear plot. For the sake of brevity, we comment on the latter plot only. It clearly consists of two segments: The upper right segment has an almost constant p/p_max_, while the segment to the left of the vertical line shows a decrease of p/p_max_ by ~five orders of magnitude. The feature of the plot in Fig. 8 (lower) is strikingly reminiscent of the one obtained by Bak *et al*.13) on different grounds within the frame of the “unified scaling law for earthquakes”, using earthquakes in California only (see their relevant Fig. 4). The origin of this similarity is currently investigated in detail.

## Conclusion

By analysing the time-series of earthquakes in the natural time-domain, we find that the power spectrum of regional and long term seismicity in Japan, exhibits features of *critical* phenomena similar to those earlier found in Greece and the San Andreas faults system. All the power spectra in these three areas fall on a *universal* curve if an appropriate reduction has been made. Furthermore, a preliminary study in the natural time-domain of the seismicities that preceded the Kobe earthquake and Off Tokachi earthquake indicates that a *critical* state is approached just a few days before the occurrence of the corresponding main-shocks.
